# Development and Assessment of the Scale of Personal Trust and Connections (PerTC): Preliminary Data from a Hospital Employee Group

**DOI:** 10.3390/healthcare11010013

**Published:** 2022-12-21

**Authors:** Emmanouil K. Symvoulakis, Panagiotis Volkos, Apostolos Kamekis, Konstantina Merou, Georgios Rachiotis, Myfanwy Morgan, Manolis Linardakis

**Affiliations:** 1 Clinic of Social and Family Medicine, School of Medicine, University of Crete, Voutes, 71003 Heraklion, Greece; 2 Fourth Local Health Team-Academic Unit of Heraklion, 71303 Heraklion, Greece; 3 School of Medicine, University of Crete, Voutes, 71003 Heraklion, Greece; 4 General Hospital of Agios Nikolaos, 72100 Agios Nikolaos, Greece; 5 Department of Hygiene and Epidemiology, Medical Faculty, School of Health Science, University of Thessaly, 41334 Larissa, Greece; 6 School of Cancer & Pharmaceutical Sciences, King’s College London, London WC2R 2LS, UK; 7 Department of Social Medicine, School of Medicine, University of Crete, Voutes, 71003 Heraklion, Greece

**Keywords:** scale assessment, healthcare professionals, trust, partnership, psychometrics

## Abstract

Trust and empathy constitute basic elements of healthcare delivery. In recent years, the quest for greater efficiency in healthcare has also indicated the necessity of these values. The study aims to develop and assess a 10-item tool, namely, the Personal Trust and Connections (PerTC) scale. The study was conducted at a general hospital in eastern Crete, Greece. A total of 218 healthcare professionals participated over a six-week period in 2021. The 10-item PerTC scale encompasses emotional, social, and cognitive reliance variables. The scale was tested for reliability, and scale scores were assessed for convergent validity. PerTC scale was found with high internal consistency (Cronbach’s α = 0.863). At a multivariate level, younger age (*p* = 0.016), more work experience years (*p* = 0.001), the experience of a recent family crisis event (*p* = 0.028), and use of the internet in free time (*p* = 0.028) were significantly related to increased total scores of the PerTC scale. The new scale is an easy-to-use metric tool with good overall reliability. PerTC may be a suitable instrument to indirectly identify determinants and drivers in order to explore pathways to collectively build on trustful interaction and altruistic connection within a healthcare environment.

## 1. Introduction

Trust has been seen as a multi-layered perception principally involving aspects of cognition based on coherent and influential rulings and emotional content shaped by relations and sentimental ties caused by communication, understanding, and similarity with others [1,2,3]. Moreover, Bell et al. (2019) meaningfully summarised that Rousseau provided an additional aspect to trust, including an inevitable acceptance of vulnerability related to optimistic expectations of the well-intentioned behavioural responsiveness of another [4,5]. The level of vulnerability is likely to be influenced by the damage and benefit summation through trust, in terms of disloyalty or loyalty returns when a trustee mutually interacts [4]. Trust appears to somehow buffer one’s uncertainty due to the risk of exposure because of the dependence on another person [2,3,6].

Granovetter (1973) argued that ‘weaker’ interpersonal ties also have an important function in providing trusted information and contacts that increase community integration and assist in accessing the required healthcare and other local resources [7]. This has informed community engagement approaches to increase the uptake of screening and other services among hard-to-reach groups (e.g., ethnic minorities), with effective local champions being both well connected in the community and trusted [8,9]. Moreover, the literature shows that trust tends to be influenced by different socio-demographic variables including education. According to Li and Fung (2013), more aged persons were significantly related to a greater perception of generalized trust and an oriented feeling of trust toward familiars [10]. Education may expand the capacity for managing information, which may enhance their social trust [11,12]. Charron and Rothstein (2016) reported that individuals with higher education tend to be more trustful [12]. Furthermore, the literature reports that persons married or living together feel more beloved and more trustful in general and have more wellness in their lives [13,14]. Trust has also been shown to be associated with gender, with women showing lower self-reported generalised trust than men to other people [14,15].

In the healthcare framework, the interaction between the trustee and the trustor requires trustworthiness, morality, discretion, affection, and respect [3,16,17], while ensuring confidence in competence may also include both practical and interpersonal bonding skills. In this relationship between hope and competence, the trustor and trustee are invited to make a joint effort in their best interests [3]. Moreover, vulnerability increases when one is ill and may unambiguously drive trust in the medical environment to become a more robust affective ingredient [3,16].

Regardless of the clinical setting, trust between health professionals in the same or different fields is vital [18]. Moreover, improving trustful beliefs and faithful cooperation between healthcare workers should enhance team spirit and guarantee better clinical results [19,20,21]. However, there are also important questions regarding the readiness and ability of individuals, whether doctors or patients, to build interpersonal connections and achieve trustful relationships. Therefore, we aimed to develop a scale that could be used effortlessly in a consulting room in order to approach persons with a limited tendency to build trustful connections who possibly need more attention as citizens, professionals, students, or patients. We also hypothesized that assessing the properties of a measurement tool in a healthcare environment offers an opportunity to better deal with all aspects that encompass trust issues such as emotions, cognitive experience, and social support. This study aims to create and assess the Personal Trust and Connections (PerTC) scale, a tool that attempts to quantify an individual’s inclination and ability to achieve trusting relationships.

## 2. Materials and Methods

### 2.1. Setting and Sampling

This study used a quantitative prospective methodology to collect the necessary data. The study population comprised healthcare providers (doctors, nurses, and other healthcare workers) working at the General Hospital of Agios Nicolaos in Lasithi, Crete, Greece. The hospital is part of the National Health System and covers an area of 75,381 inhabitants in the eastern part of the island, which relies mainly on tourism, agriculture, and livestock. In addition to secondary in-patient care, the hospital provides emergency triage management and outpatient clinic care. A convenience sampling design was used. In total, 250 questionnaires were distributed, and 218 healthcare employees participated in the six-week data collection period (from May until June 2021). Respondents comprised approximately 70% of the total number of healthcare professionals employed during the study period.

### 2.2. Ethics Approval

The approval to conduct a study for the needs of an MSc thesis on daily life limitations and stress among hospital providers during the pandemic was obtained from the Scientific Committee of the General Hospital of Agios Nikolaos, Lasithi, Crete (Protocol No: 122/05-11-2020), and additional permission to include and assess the current scale was also given by the same Committee (Protocol No: 38/11-05-2021). Anonymous completion of the questionnaire was considered equivalent to informed consent.

### 2.3. Structural Content of the Questionnaire

The structure of the questionnaire was based on the Personal Sociability and Connections Scale (PeSCS) by Symvoulakis et al. (2021) [22]. The aim was to design an instrument measuring an individual’s tendency for trustful connections in everyday life. A preliminary version of the questionnaire, namely PerTC, was drafted based on a literature search ‘after a narrative conceptualization’ of the content. It was pivotally assessed by asking two people to offer responses, recommend further items, and identify any overlapping or otherwise redundant items. The necessary adjustments were made, and the ultimate version of the tool was produced. It contains 10 short questions (items) with responses on a 10-point Likert-type scale (from 0: not at all, to 10: very much): 1. Can you trust persons you have met for a few times? 2. Do you rapidly feel close to persons you trust? 3. Do you easily talk about personal matters with persons you trust? 4. Do you prefer to spend your free time alone? 5. Do you seek friendship only with persons you trust? 6. Do you easily trust persons you admire? 7. Can you easily understand the reasoning of persons you trust? 8. Do you seek trust in your social contacts? 9. Do you easily trust persons who inspire you? 10. Do you usually agree with persons you trust? Scoring for question four was reversed for meaning conformity reasons.

### 2.4. Statistical Evaluation

Exploratory factor analysis (EFA) was performed to assess structural validity. Principal component analysis (PCA) and varimax rotation were applied, with Kaiser normalization as the rotation method (oblique). The Kaiser–Meyer–Olkin (ΚΜO) measure of sampling appropriateness was assessed at 0.847 (meritorious fit), and Bartlett’s test of sphericity was adopted to check the dataset suitability for factor analysis, confirming the level of interconnection between the items (χ2 = 888.2, df = 45, *p* < 0.001). Three componential groups/factors were distinguished with eigenvalues ≥ 1.00 and 66.86% interpretation of total variance. A minimum loading criterion of 0.50 was accepted for scale items to meet the inclusion in each factor. In summary, the 10 questions were clustered into three componential groups: (i) The Emotional Reliance componential factor related to feelings shaping confidence (items one, two, six, nine, and ten); (ii) The Social Reliance componential factor related to elements shaping social connections (items three, five, and eight); and (iii) Cognitive Reliance componential factor leading to determinants shaping rational meanings of confidence (items four and seven). It is noted that for the PerTC scale, a test–retest reliability procedure was conducted by 16 participants, with a completion intermediate time of two weeks.

To assess the scores for the aforementioned componential groups, as well as for the overall score of the PerTC scale, the relevant item answers were averaged, leading to a variable range in composite scoring. Data from the test–retest reliability showed that, within the two weeks between phases one and two, r-Pearson = 0.899 (*p* < 0.001) was estimated for the total score, with r = 0.874 (*p* < 0.001) for the Emotional Reliance, r = 0.916 (*p* < 0.001) for the Social Reliance, and r =0.891 (*p* < 0.001) for the Cognitive Reliance (‘good-to-excellent reliability’).

### 2.5. Statistics

The analysis was conducted using SPSS software (IBM Corp. Released 2019, IBM SPSS Statistics for Windows, v.26.0, Armonk, NY, USA: IBM Corp.). Allocations of frequency in regard to descriptive and other features of the 218 enrolled participants were assessed, with the respective 95% confidence intervals for the purposes of comparison. The form of allocations of the PerTC scores, as well as of the scores of its three componential factors, was tested using Blom’s method (QQ plot), and their reliability coefficients were calculated using Cronbach’s method. Due to slight asymmetry, the parametric Pearson’s method either between the componential factors (also as an indication of convergent validity) or against the participants’ characteristics was used. The regression coefficients (β, unstandardized beta) were calculated through multiple linear regressions for the correlation between the PerTC scale and the participants’ demographic characteristics, or the recent tragic events in their lives and general internet use. The accepted materiality level was set to 0.05.

## 3. Results

### 3.1. Demographics

Of the 218 study participants, 72.9% were women. Respondents’ age, on average, was 43.2 years (±10.5), 59.2% were married, and only 9.6% were not cohabitating. Altogether, 9.6% were holders of an MSc or PhD, and the average number of ‘occupational years’ was 14.8 years (±10.5). On average, they had the perception of being eight years younger than they actually are, while 29.4% felt as old as they really are or more aged than they actually are (Table 1).

### 3.2. Occurrence of PerTC Scale Responses

As shown in Table 2, the sum of very positive responses (eight, nine, and ten points), was most frequently reported for question eight, ‘Do you seek trust in your social contacts?’, with 47.3% of the participants fully in agreement, followed by item five, ‘Do you seek friendship only with persons you trust?’, with 30.8% of fully positive answers. in contrast, the sum of very negative responses (zero, one and two points), was the most commonly preferred response to item one, ‘Can you trust persons you have met for a few times?’, with 57.4% of the participants fully in disagreement, followed by question four, ‘Do you prefer to spend your free time alone?’, with 42.1% of respondents responding negatively.

### 3.3. PerTC Scale Scoring

The average score of the PerTC scale was 5.29 points (±1.41), for the Emotional Reliance componential factor, representing the expression of feelings shaping confidence (questions one, two, six, nine, and ten), (Table 3), 4.89 (±1.59), for the Social Reliance componential factor related to elements shaping social connections (questions three, five, and eight) 6.34 (±1.58), and 4.74 (±2.04) for the Cognitive Reliance componential factor leading to determinants shaping rational meanings of confidence (questions four and seven). The PerTC scale was ranked ‘meritorious’ (Cronbach’s alpha: 0.863), as was the Emotional Reliance componential factor (Cronbach’s alpha: 0.838). The Cognitive Reliance componential factor was ‘acceptable’ (Cronbach’s alpha: 0.761), and the Social Reliance componential factor had a Cronbach’s alpha of 0.657. The association between the three componential groups and the entire PerTC scale scores (Table 4) supported their convergent validity, with a significant association (*p* < 0.001). Table 5 shows the univariate regression analysis of PerTC scale componential factors and scores, and the 218 respondents’ features. In the univariate-level analysis, occupation years (*p* < 0.05) and recent experience of a dramatic event in their family (*p* < 0.05) seem to be significantly related to increased PerTC scale total scores.

Table 6 provides information on the multiple linear regression analysis performed. At a multivariate level, younger age (β = −0.04, *p* = 0.016) and more occupational years (β = 0.06, *p* = 0.001) seem to be significantly related to increased PerTC scale total score. Furthermore, those who experienced a recent dramatic event in their family (β = 0.51, *p* = 0.028) and those who use the internet in their free time offered responses with significantly higher PerTC scale scores compared to their peers (β = 0.63, *p* = 0.028).

## 4. Discussion

The present study delivers an exploratory assessment of a newly developed scale to measure an individual’s propensity to cultivate trustful interactions in everyday life. Five out of ten participants seek trust in their social contacts. Approximately three out of ten respondents seek friendship only with persons they trust. Score levels and the reliability of each scale domain (emotional, social, and cognitive), with Emotional Reliance contributing a consistent Cronbach alpha (0.838), are shown in Table 3. Affective influential determinants shaping the need for trust, the promptness to offer care, and the perception that these emotions are shared and returned may explain, at least partially, a two-sided beneficial bond [3,23]. Another instrument measuring interpersonal trust, the Korean version of the Specific Interpersonal Trust Scale, reported partially similar componential groups, classified as ‘overall trust’, ‘emotional trust’, and ‘reliableness’ [24].

Our survey showed that age was slightly inversely related to trust among the study participants. Previous research suggested that, among older persons, trust is enhanced in order to maintain connectedness [10,25]. Moreover, our study showed that more working years were positively related to trust. An explanation for this outcome may be that more qualified counterparts are likely to be less prone to depend on less skilled associates than the opposite [26]. From the univariate analysis (Table 5), we found that the cognitive domain interacts with the total PerTC scale score in regard to its correlation with years of working experience.

Previous studies presented links between connectedness, well-being, job gratification, decision management, and working achievements [27,28,29,30]. People are thought to strive for relationships in which both sides trust each other since the absence of this balance can cause a sense of uncertainty [30,31] and, in support of this, previous studies report mutuality as an important element within relationships [30,32]. Our results show that a recent experience of a dramatic event in the family seems to be significantly correlated with increased PerTC scale scoring. According to Bell et al. (2019), those who had experienced a dramatic event and had the ability to use feedback to modify their behavioural output presented a reduced tendency to cooperatively trust others in comparison with controls [4]. This could be related to limited trust in oneself [4]. Participants, in our study, work in a setting where dramatic events are seen with a more altruistic and social support manner and perhaps this lived experience is inversely translated when projected at a personal level of psycho-social defence. From the univariate analysis (Table 5), we know that both domains, emotional and social, interact somehow with the total PerTC score and its correlation with the dramatic event as variable. Moreover, participants who used the internet in their free time had significantly higher levels of the total PerTC scale. In the era of the internet, people progressively perform social contact online or interact through e-communication [33]. A finding that emerged from the multivariate analysis of our study shows that internet use in free time positively correlates with increased levels of trust among the participants.

Future research could focus on a variety of parameters associated with increased trust in relation to healthcare issues, services, and outcomes (e.g., greater patient satisfaction, and better adherence to treatment) [34,35,36]. Shifting from old-fashioned meanings of health service structuring to an equal partnership and respectful interactions may be helped by improving patients’ trust in healthcare professionals by evolving medical communication skills toward patient centeredness, enhancing confidentiality by consensual agreements, and improving access to care with doctors who show familiarity and availability [37]. Additionally, the PerTC scale may interact with empathy. Regardless of whether empathy is perceived as a virtue or, for some, as a skill, the healthcare environment is always the natural field for its cultivation or teaching [34,38]. Greater empathy can be a protective factor for health professionals against work-related suffering and burn-out and can support them to interact with their patients effectively and altruistically [34,39,40]. The PerTC scale may help to highlight whether empathy temperament, when linked to future research findings, is related to propensity and promptness for trust in a healthcare working environment. Moreover, a study in Italy reported that some healthcare professionals were unwilling to report sentinel events to clinical risk management hospital service because they feared possible blaming consequences [41]. Although the purpose of event reporting was to avoid future similar errors, scepticism was the determinant of their decision [41]. Several implications may occur in daily routines as trust is a common ingredient in all interpersonal situations. PerTC may measure to what extent the lack of trust is related to an individual’s tendency to think and act in terms of sincere motivation, driven by trustful professional relationships. Another interesting aspect would be to study the effect of PerTC on participation and teamwork ability trends in the context of group professionals or collective sports activities [22,42] as further practical implications of this work.

However, researchers or other professionals should be careful when choosing a tool to measure trust. The study of Dai et al. (2020) compared three widely used scales, namely, the Interpersonal Trust Scale (ITS), the Philosophies of Human Nature Scale (RPHNS), and the Company Trust Scale (CTS) in the Chinese cultural context and concluded that when measuring trust, the researcher or professional should take into consideration the two major trust groups: General (which refers to society or institutions) and special (which refers to family members, acquaintances, etc.) trust [43]. The cultural background should also be taken into account [43].

### Limitations

This study was based on secondary-care hospital personnel in Crete and a single cohort of healthcare employees living in an urbanised location on a touristic island. We are therefore not able to generalise the prevalence of particular attitudes and feelings measured by the scale to different contexts or geographical settings elsewhere. The study was cross-sectional with self-completing questionnaires and we do not yet possess data on how levels of trust, detected early with our scale, evolve over time or during a lifetime. Data were also collected during a pandemic, and we cannot predict how a situation of social distancing or working pressure in a healthcare environment altered the views or responses in matters that deal with social connections and related feelings. Furthermore, it would be interesting to test whether longitudinal data should be gathered to examine the stability of the measurement scale. Future research should employ a confirmatory factor analysis using structural equation modelling.

## 5. Conclusions

This study developed and validated a scale to assess personal trust and connections. The scale is composed of 10 items with high overall reliability and may be used in assessing the ability to develop trustful interconnection with measures including empathic experiences and to easily recognize those persons who may benefit from possessing specific ‘skills’. In summary, this study validated a measure of propensity to trust, and we hope that future efforts will be made to examine the dispositional component of trust in everyday decisions.

## Figures and Tables

**Table 1:** Basic descriptive characteristics of 218 study participants.

		n	%
Gender	male/female	59/159	27.1/72.9
Age, years	mean ± stand. dev. (min, max)	43.2 ± 10.5 (20, 67)	
Family status	married/unmarried, divorced, widow	129/89	59.2/40.8
Household size, persons	None	21	9.6
	1	39	17.9
	2	42	19.3
	3+	116	53.2
Education	High School education	28	12.8
	Technical education	86	39.4
	Higher Education	83	38.1
	MSc, PhD education	21	9.6
Occupation, years	mean ± stand. dev. (min, max)	14.8 ± 10.5 (1, 40)	
	Permanent employment	90	41.3
	Fixed-term employment	79	36.2
	Practicing or labour market opportunity contract	14	6.4
	External Associate	2	0.9
	Other Not defined	14 19	6.4 8.8
Sense of age, years-	mean ± stand. dev. (min, max)	35.4 ± 12.7 (10, 90)	
Δ-difference from age	mean ± stand. dev. (min, max)	−7.8 ± 11.3 (−42, 49)	
	Δ ≥ 0.00 (feel older)	63	29.4

**Table 2:** Frequency responses and mean levels of 218 participants on ‘Scale of Personal Trust and Connections, PerTC’ items.

	Responses											
	0	1	2	3	4	5	6	7	8	9	10	Mean
	Not at All								Very Much			
	%											
1. Can you trust persons you have met for a few times?	12.4	17.0	28.0	12.4	6.9	10.6	4.1	3.7	4.1	0.9	0.0	2.82
2. Do you rapidly feel close to persons you trust?	0.9	3.7	6.9	9.2	13.8	24.8	10.6	12.8	11.0	4.6	1.8	5.25
3. Do you easily talk about personal matters with persons you trust?	2.3	2.3	3.7	8.3	16.1	25.7	12.8	11.0	10.6	4.1	3.2	5.35
4. Do you prefer to spend your free time alone?	6.4	15.1	20.6	14.2	5.0	14.2	6.9	5.5	6.9	2.8	2.3	3.73
5. Do you seek friendship only with persons you trust?	0.0	0.0	1.4	3.2	11.9	23.4	16.1	13.3	12.4	10.6	7.8	6.39
6. Do you easily trust persons you admire?	1.8	2.8	3.2	11.5	15.6	32.1	12.8	9.2	6.9	2.8	1.4	5.01
7. Can you easily understand the reasoning of persons you trust?	0.0	0.5	1.8	11.5	17.0	21.1	13.3	11.0	12.4	6.9	4.6	5.76
8. Do you seek trust in your social contacts?	1.4	0.0	1.4	1.8	1.8	10.1	12.8	23.4	17.9	14.7	14.7	7.29
9. Do you easily trust persons who inspire you?	1.8	0.9	2.3	6.0	15.6	24.8	16.5	13.8	10.6	5.0	2.8	5.62
10. Do you usually agree with persons you trust?	0.9	0.9	2.3	4.6	11.0	32.6	17.0	12.8	9.2	5.5	3.2	7.73

**Table 3:** Score levels and reliability of ‘Scale of Personal Trust and Connections, PerTC’.

Factors/Components	Mean	Stand. Dev.	Median	Min	Max	Cronbach’s α
Emotional Reliance (Questions 1, 2, 6, 9, and 10)	4.89	1.59	4.60	0.0	9.2	0.838
Social Reliance (Questions 3, 5, and 8)	6.34	1.58	6.00	1.7	10.0	0.657
Cognitive Reliance (Questions 4 and 7)	4.74	2.04	4.50	1.0	10.0	0.761
Total Personal Trust and Connections Scale score	5.29	1.41	5.00	1.4	9.0	0.863

**Table 4:** Correlations between components and ‘Scale of Personal Trust and connections, PerTC’ scores of 218 participants in the current study.

	Total Personal Trust and Connections Scale	Emotional Reliance	Social Reliance
	r-Pearson (*p*-value)		
Emotional Reliance	0.919 (<0.001)		
Social Reliance	0.807 (<0.001)	0.611 (<0.001)	
Cognitive Reliance	0.732 (<0.001)	0.523 (<0.001)	0.442 (<0.001)

**Table 5:** Univariate correlations of the ‘Scale of Personal Trust and Connections’ and its components with the characteristics of the 218 study participants.

	Total Personal Trust and Connections Scale Score	Emotional Reliance	Social Reliance	Cognitive Reliance
	r-Pearson			
Gender (1:male, 2:female)	0.100	0.067	0.140 *	0.053
Age (years)	0.035	0.030	0.009	0.053
Family status (1: married, 2: unmarried, divorced, widow)	−0.081	−0.104	−0.007	−0.070
Household size	0.074	0.100	0.004	0.057
Education (1: High School education, 2: Technical education, 3: Higher Education, 4: MSc, PhD education)	−0.035	−0.054	0.016	−0.034
Occupation (years)	0.142 *	0.122	0.094	0.142 *
Sense of age (yrs, Δ-difference from age)	0.036	−0.015	0.075	0.064
Recent dramatic event in family (1: no, 2: yes)	0.156 *	0.147 *	0.169 *	0.057
Internet use in free time (1: no, 2: yes)	0.097	0.126	0.059	0.021

* *p*-value < 0.05.

**Table 6:** Multiple linear regression analysis of ‘Scale of Personal Trust and Connections’ scores and the characteristics of 218 study participants.

	Total Personal Trust and Connections Scale Score			
Parameters	β	95% CI		*p*-Value
Gender (1:male, 2:female)	0.34	−0.08	0.76	0.113
Age (years)	−0.04	−0.08	−0.01	0.016
Family status (1: married, 2: unmarried, divorced, widow)	−0.05	−0.52	0.41	0.821
Household size	0.07	−0.09	0.24	0.389
Education (1: High School education, 2: Technical education, 3: Higher Education, 4: MSc, PhD education)	−0.09	−0.33	0.15	0.473
Occupation (years)	0.06	0.02	0.09	0.001
Sense of age (yrs, Δ-difference from age)	−0.01	−0.02	0.01	0.690
Recent dramatic event in family (1: no, 2: yes)	0.51	0.06	0.96	0.028
Internet use in free time (1: no, 2: yes)	0.63	0.07	1.20	0.028
R^2^ (adjusted)	0.109 (0.062)			

## Data Availability

Datasets are available from the first author upon reasonable request.
